# Novel Anti-Cancer Agents and Cellular Targets and Their Mechanism(s) of Action

**DOI:** 10.3390/biomedicines10081767

**Published:** 2022-07-22

**Authors:** Simon J. Allison

**Affiliations:** School of Applied Sciences, University of Huddersfield, Huddersfield HD1 3DH, UK; s.allison@hud.ac.uk; Tel.: +44-(0)1484-256857

Whilst there have been some significant improvements in treatments and patient outcomes for some cancers, for other cancers there has been little change in survival rates for many years. Side effects resulting from the toxicity of agents towards normal cells and tissues and the development of drug resistance continue to limit the effectiveness of traditional and molecular-targeted anti-cancer agents. Other challenges include pharmacokinetics (PK) and delivering sufficient amounts of the active drug to tumour cells in vivo. There is an ongoing need for new anti-cancer agents with novel mechanism(s) of action and the identification of new putative cellular targets and therapeutic strategies that are both potent and selective towards cancer cells.

Addressing some of these therapeutic challenges, this book presents a collection of eight research articles and two reviews reproduced from the themed *Biomedicines* Special Issue ‘Novel Anti-Cancer Agents and Cellular Targets and Their Mechanism(s) of Action’.

Traditional cytotoxic chemotherapeutic agents remain a vital part of treatments for many cancers, but their efficacy is severely restricted by their lack of selectivity and dose-limiting toxicity towards healthy cells and tissue. Affibody or antibody drug conjugates provide an opportunity to target or deliver cytotoxic agents specifically to cancer cells, exploiting a tumour-specific antigen specifically recognised by the conjugate for targeted delivery. Rinne et al. report on a human epidermal growth factor receptor 3 (HER3) affibody drug conjugate with a tubulin polymerisation inhibitor as its cytotoxic ‘payload’ for targeted delivery to HER3-overexpressing cancer cells [1]. The authors also include an albumin binding domain as part of the conjugate to improve its half-life and PK properties [1].

A different approach to using affibody drug conjugates to selectively target cancer cell microtubules is presented by Kwon and colleagues [2]. In triple-negative breast cancers (TNBC), which have the worst outcome of the different breast cancer subtypes, microtubules are found to be highly acetylated. Kwon et al. report on their identification of several compounds that disrupt microtubule acetylation in TNBC cells resulting in their apoptotic demise [2].

One cancer type with particularly poor outcome is glioblastoma (GBM), which has a five-year median patient survival rate of less than 5%. The invasion of cancer cells into healthy brain tissue makes tumour resection particularly challenging, resulting in high rates of tumour recurrence. This has stimulated interest in pharmacological inhibition of cell migration as part of the regime for treating glioblastomas. Ketchen et al. report on the migratory plasticity of GBM cells and how the pharmacological inhibition of mesenchymal migration, via the inhibition of cellular communication network factor 1 (CCN1), promotes cells to ‘switch’ to an alternative means of migration known as ameboid migration [3]. This work indicates the importance of simultaneously targeting multiple alternative modes of GBM cell migration.

With our increased understanding of cancer biology and of oncogene-driven molecular addictions, this has resulted in considerable research effort to exploit and target these addictions within specific cancers via molecular targeted anti-cancer drugs and through rational drug design approaches. However, it is also clear that whilst such agents can present important advantages over traditional agents such as their increased cancer selectivity, other challenges remain such as the ability of cancer cells to adapt and develop resistance. This concept of some targeted agents being perhaps ‘too targeted’ has led to a resurgence of interest in the discovery of novel chemical entities with polypharmacological anti-cancer properties and in phenotypic drug discovery. The natural world continues to be a valuable source of novel chemicals with anti-cancer activity. Eldeeb et al. report on their testing of ten novel isatin sulfonamide derivatives for anti-cancer activity against hepatic cancer cell lines and associated in vitro mode of action and molecular docking studies [4].

A major challenge in phenotypic drug discovery and development is that of target deconvolution. Wu et al. report on an isoflavone derivative from fermented soybean, 8-hydroxydaidzein, which shows promising activity against chronic myeloid leukemia (CML) cells, with the authors reporting effects via multiple mechanisms of action [5]. Nikoleousakos et al. report on the synthesis of three novel β-lactam steroid alkylators and their in vitro evaluation against human ovarian cancer cell lines. As well as DNA damage induction, the inhibition of poly (ADP-ribose) polymerase (PARP) enzymatic activity is reported [6].

A major cause of treatment failure in vivo is the metabolic detoxification or inactivation of anti-cancer drugs by isoforms of the cytochrome P450 (CYP) family of enzymes. In other cases, the activity of specific CYP isoforms can result in the generation of more active, or cytotoxic, metabolites. Pors and colleagues review the role of CYP isoforms in breast cancers and how they influence different standard-of-care (SoC) treatments [7]. Importantly, they also discuss new therapeutic opportunities presented by the overexpression of particular CYP isoforms in different breast cancer subtypes, which is stimulating the development of CYP isoform-activated pro-drugs [7]. This tackles the challenge of developing drugs that are both potent and also tumour-selective, in contrast to the narrow therapeutic index of traditional chemotherapies.

For molecular targets in oncology that are considered ‘undruggable’, or perhaps more accurately ‘difficult to drug’, a potential solution to this druggability problem is RNA interference (RNAi)-based therapeutics. A key barrier to this latter approach, however, is the susceptibility of short interfering RNA (siRNA) to degradation in vivo by nucleases in serum. Approaches to counteract this and improve siRNA stability include chemical RNA modifications or siRNA extension with a ‘protective’ short DNA hairpin loop sequence. Another approach attracting considerable research interest is siRNA delivery within biodegradable nanoparticles which is discussed by Habib, Ariatti and Singh in their review article focused on RNAi-based nanotherapeutics in the context of the ‘undruggable’ *c-myc* oncogene [8].

A challenge with any anti-cancer agent is ensuring its sufficient delivery to the tumour in vivo. Poor in vivo biodistribution/PK often contributes to the in vivo failure of otherwise promising drugs. The ability to ‘visualise’ drug delivery in vivo provides the opportunity to assess whether this is an issue and for developing more effective drug delivery systems. Vepris et al. report on their development of novel poly(lactic-co-glycolic acid) (PGLA) fluorescent nanoparticles as delivery agents that enable in vivo molecular imaging through the phenomenon of photon triplet-triplet annihilation (TTA) upconversion [9].

The final article in this Special Issue book focuses on 5-fluorouracil (5-FU) drug resistance in colorectal cancers (CRC), where 5-FU is part of the standard-of-care, and pharmacogenomic analyses using publicly available resources to predict agents that can overcome 5-FU resistance depending on the particular CRC mutational signature [10].

In summary, this Special Issue collection of articles provides some examples of the many different approaches being used to tackle the multiple challenges that remain in the global battle against cancers. These range from the evaluation of novel compounds for anti-cancer activity and elucidation of their mechanism(s) of action to overcoming drug resistance and advances in targeted drug delivery and molecular imaging for the validation of effective delivery.
